# Six Sigma Application in Healthcare Logistics: A Framework and A Case Study

**DOI:** 10.1155/2019/9691568

**Published:** 2019-02-14

**Authors:** Lina Al-Qatawneh, Abdallah A. A. Abdallah, Salam S. Z. Zalloum

**Affiliations:** ^1^Department of Industrial Engineering, The University of Jordan, Amman, Jordan; ^2^Graduate School of Business Administration, The German Jordanian University, Amman, Jordan; ^3^Department of Project Management, Incube Mobility Solutions, Amman, Jordan

## Abstract

Six Sigma is used heavily in various industrial sectors, yet no noticeable applications are seen in healthcare logistics. This paper reveals the special case of healthcare logistics where cost reduction is not the only factor considered in project selection; performance and criticality of each item in the logistics system are of high importance as well. This paper provides a proposed framework to apply Six Sigma in the area of healthcare logistics. It also presents a case study implementing the proposed framework at a Jordanian hospital. In the case study, the paper reveals how the modifications of the define phase to take into consideration the criticality, cost, and performance of items make typical Six Sigma methodology very beneficial for healthcare logistics. In addition, it shows how the Six Sigma project selection can be done to deal effectively with healthcare logistics issues. This paper paves the road for research to elaborate on ways to use Six Sigma in the area of improving healthcare logistics.

## 1. Introduction

Hospitals generally think of their offerings as services rather than products. The core service is patient care. However, the provision of medical treatment and patient care creates demand for tangible medical and nonmedical products. Although personnel, nursing, and physician pay accounts for a large portion of a hospital's operating budget, yet costs related to inventory, logistics, and administration processes are nevertheless significant. Studies [1–3] have shown that 30% to 40% of hospital spending is invested in various logistical activities, such that approximately half of this amount derives from the direct cost of acquiring materials and services and the other half from the cost of managing them after acquisition. Nowadays, healthcare providers are seeking to improve their logistics and supply chain management in order to reduce the current high healthcare cost.

Literature offers many studies about logistics in healthcare. Jørgensen et al. [4], Ferretti et al. [5], and Volland et al. [6] revealed several focus areas in the healthcare logistics' studies including logistics activities (supply and procurement, inventory management, transportation, and distribution and scheduling), holistic supply chain management, lean logistics, patients' logistics, and logistics technology. In the search for ways to improve healthcare logistics, academics and practitioners have looked into methodologies that have been applied successfully in other sectors, especially the manufacturing sector. Although Six Sigma has been applied successfully in the manufacturing sector as reported in a recent literature review study [7], the methodology is less frequently applied in healthcare logistics. Limited research has evaluated whether the Six Sigma methodology transfers successfully and what impact the methodology has on for example productivity, costs, and quality of service. This paper makes two contributions. First, this research proposes how Six Sigma can be employed to improve healthcare logistics. Secondly, this paper introduces a novel approach to determine the critical Six Sigma projects which provide maximum benefits to the healthcare organization. The approach is based on defining Importance Index that correlates criticality, cost, and performance of products in healthcare logistics.

This paper is organized as follows.  Section 2 provides a literature review of Six Sigma and its application in healthcare logistics, followed by a literature review of what healthcare logistics involves. In Section 3, the proposed framework for applying Six Sigma in healthcare logistics is presented in detail. In Section 4, an empirical case study from healthcare is given to explore the effectiveness of the proposed framework. In the last section, the findings of this research are discussed.

## 2. Literature Review

### 2.1. Six Sigma

Six Sigma was originally developed in the mid-1980s by Motorola as a quality control method to prevent defects in their manufacturing process [8] and has been evolved into a project-driven management approach to improve the organization's products, services, and processes by continually reducing defects in the organization [9]. Six Sigma is defined in a variety of ways by several authors. From a statistical perspective, Six Sigma is defined by Motorola as a quality improvement program with a goal of reducing the number of defects to as low as 3.4 parts per million opportunities [10]. From a business perspective, Six Sigma is defined as a business strategy used to improve business profitability and to improve the effectiveness and efficiency of all operations to meet or exceed the customer's needs and expectations [11].

Zu et al. [12] identified three practices that are critically associated with Six Sigma implementation: Six Sigma role structure, Six Sigma structured improvement procedure, and Six Sigma focus on metrics. The role structure of Six Sigma is often referred to as the “belt system.” Six Sigma identifies several key roles for its improvement specialists: champions, master black belts, black belts, and green belts [13, 14]. Those specialists are assigned different levels of roles and responsibility and work together in a hierarchical coordinated mechanism across multiple organizational levels to achieve the Six Sigma goals.

Six Sigma uses two major structured methods for improvement known as DMAIC (define-measure-analyse-improve-control) and DMADV (define-measure-analyse-design-verify). The DMAIC method is used for process improvement [15], while the DMADV is used for product and process design [16]. The highlight of the DMAIC method is the five-phased methodological framework that guides in conducting the improvement project [17, 18]. In the first four phases, various managerial and statistical tools are used in a manner that makes it easy to understand the process and its issues as well as finding the proper root causes of the problem and coming up with the proper solutions. The last phase ensures that the root causes of the problem seize to exist and the process will never go back to its old ill state.

### 2.2. Six Sigma Application in Healthcare Logistics

The application of Six Sigma in healthcare logistics had very little attention in practice. Jin et al. [19] presented a detailed case study of applying the principles and procedures of Six Sigma and Lean thinking in designing and operating a healthcare logistics centre in North Mississippi. Craven et al. [20], through brief case studies, highlighted some representative Six Sigma projects conducted in various departments at New York–Presbyterian hospital. Their study described how an inventory management project that used Lean Six Sigma methodology resulted in the identification and removal of expired medication and products. Lifvergren et al. [21] described the lessons learned from 22 Six Sigma projects implemented by a Swedish hospital—two of which were related to logistics. Their study provided a summary of the project purpose, whether the project reached its intended results and the net cost savings in the first year after the implementation of the suggested solutions. A critical analysis overview of the important publications is presented in Table 1.

### 2.3. Healthcare Logistics

In healthcare, logistics systems are complex and more problematic to manage compared to other sectors. This is due to the wide product range, the high value of products involved, and the perceived need to supply a very high level of services for most items [22]. The wide variability in product ranges is caused by the high differentiation among available products, the subjective role of physicians in choosing these products [23], and most importantly, the large volume of diverse support services required to deliver the end product–patient care. Moreover, healthcare providers are unable to predict patient mix and hence unable to predict and control the demand of products [3].

Products used in hospitals can be classified into categories based on the level of criticality to patient care. Although critical items constitute a small number of items, the majority of the total inventory investment is in critical items—around 60% [24]. Critical items are usually extremely expensive, have short shelf-life, and/or require expensive storage facilities on-site. What makes hospital logistics more complex is the criticality of some items used and the patient's life-threatening situations that could happen due to the unavailability of these items in stocks. This distinctive feature of hospital logistics may require different management policies than those used for other industries logistics systems. Al‐Qatawneh and Hafeez [25] proposed a multicriterion critical-to-life classification technique for managing inventory in a healthcare supply chain. Their findings suggest that the proposed classification allows for assigning a particular service level to each item to ensure the availability of items that are critical to patient life and deduce the optimal inventory level.

The literature of Six Sigma application in healthcare logistics showed that no work is available that considers explicit interrelations between criticality of items and problem definition in a Six Sigma project. To answer this deficiency, this paper proposes the use of an Importance Index that correlates criticality, cost, and performance to select the Six Sigma project.

## 3. Proposed Framework

Cost and customer satisfaction are two key factors that are considered when taking managerial decisions by healthcare logistics professionals. The criticality level of an item affects cost and customer satisfaction in a conflicting way. For example, for an item that is deemed critical, the availability of such an item is more important than the expenses of procurement, storage, and transportation. On the other hand, cost optimization tends to minimise on-hand stock. Therefore, two competing objectives have to be satisfied in a manner that minimises the cost while guaranteeing no stock-out incidents, especially for critical items. So, for a Six Sigma project focusing on healthcare logistics, the objective can be minimising the total logistics cost while maintaining a high performance level.

When selecting the Six Sigma project, we correlate criticality, cost, and performance by defining Importance Index (II) as follows:

(1) $$\begin{array}{c} \text{Importance Index}=\left(F1\times \text{criticality level}\right)+\left(F2\times \text{cost level}\right)+\left(F3\times \text{performance level}\right), \end{array}$$

where 0 ≤ *F*1 ≤ 1, 0 ≤ *F*2 ≤ 1, and 0 ≤ *F*3 ≤ 1.

*F*1, *F*2, and *F*3 are determined for every item in the stock, depending on the gap between the current situation and the situation the hospital wants to achieve, such that the larger the gap, the higher the value of the factor *F*. The factor *F* will have a value of zero if the performance level equals its target and will have a value of one for the highest possible gap between the performance level and its target.

*Criticality level* indicates criticality of the item based on a criterion decided by the voice of the customer (VOC), and it equals 1 for noncritical items, 5 for medium critical items, and 9 for highly critical items. The estimation of the level is done twice, once by the process owners and the second by the Six Sigma team.

*Cost level* indicates the cost of the item based on the critical to quality (CTQ) parameter decided by VOC, and it equals 1 for low cost items, 5 for medium cost items, and 9 for items with high cost.

*Performance level* indicates the performance of the item based on a CTQ parameter decided by VOC, and it equals 1 for excellent performance, 5 for medium performance, and 9 for poor performance. The estimation of the level is done twice, once by the process owners and the second by the Six Sigma team.

The project with highest Importance Index (II) value will be selected. The II values can range between 0 (for a project on an item that is on target for all the three levels mentioned) and 27 (for a project on an item that has its performance characteristic at the poor end of the scale, is highly critical to patients, is a costly item, and is off target for all the three levels).

Notice that a Six Sigma project will try to decrease the value of II. The criticality value is hard to minimise, still, it can be minimised through creating alternatives, minimising the effect of stock-out, shortening the time of replenishment, etc. The performance level and cost of operation are typical Six Sigma improvement projects, where projects are conducted to reduce the gap between the current and the targeted situation.

In the improve phase when selecting between alternatives, we select the alternative that is expected to have the greatest impact on decreasing the II value after implementing the new process changes. Also, the Six Sigma project success can be measured by the amount we reduce the value of II in the project.

The intriguing part of the proposed methodology is that it can be used to deal with various areas of healthcare logistics. These areas vary from inventory control to transportation, warehousing, supplier management, customer service, and demand forecasting.

Table 2 reveals our step by step framework that can be used effectively to tackle healthcare logistics. The framework is a proposed modified Six Sigma methodology that fits healthcare and its logistics system.

## 4. Case Study

Our case study organization is a general hospital from the private sector in Jordan. Due to confidentiality, we will refer to it as the case hospital. The following sections will discuss in detail the application of the proposed framework different steps shown in Table 2 at the case hospital.

### 4.1. Define Phase

#### 4.1.1. Fully Define the Process

A clear knowledge of the case hospital's logistics system was acquired. The case hospital conducts two main logistics activities: warehousing and inventory management. The case hospital has four main warehouses: medical supplies warehouse, nonmedical supplies warehouse, maintenance warehouse, and pharmaceutical supplies warehouse. In addition to the four main warehouses, there is a secondary warehouse located at each department, which will be referred to as “department warehouse.” The department warehouse contains a one week stock of most frequently used items by the department. In this paper, the application of Six Sigma is done only on the medical supplies warehouse.

#### 4.1.2. Define the Parameters That Will Be Used to Assess the Process Performance

Determining the CTQ parameter that will be used for assessing the logistics system performance for medical supplies was based on the voice of the customer. A questionnaire was designed, and a survey was conducted which included internal customers (warehouse keepers) from the targeted warehouse, and four other secondary warehouses included emergency room (ER) department warehouse, operations room (OR) department warehouse, cardio department warehouse, and extension ward warehouse. The total number of respondents was five warehouse keepers. The survey required process owners to rank the suggested CTQs in the questionnaire as high, medium, or low in terms of importance in performance assessment. Then, a simple scale system was used for ranks, such that (5) indicated high importance, (3) medium importance, and (1) low importance.  Table 3 shows the suggested CTQs and their average rank according to the survey findings.  Table 3 shows that “average inventory level” has the highest average CTQ rank of 4.6 which makes it the most suitable CTQ to assess the logistics system performance based on VOC.

#### 4.1.3. Define the Parameters That Will Be Used to Assess the Criticality Level of Products

This step too was based on the VOC. Another questionnaire was designed, and a survey was conducted which included internal customers (doctors, nurses, and warehouse keepers) from different departments; this survey had a total of 16 respondents. The survey required customers to rank the suggested criteria in the questionnaire as high, medium, or low in terms of suitability for criticality assessment. Then, a simple scale system was used for ranks, such that (5) indicated high suitability, (3) medium suitability, and (1) low suitability.  Table 4 shows suggested criteria and their average rank according to the survey findings.  Table 4 shows that both “time needed to get product from the nearest distributor warehouse” criterion and “effect of stock-out incidents or problem caused by stock-out condition” criterion have the highest average rank of 4.1 which makes them the most suitable criteria for assessing the criticality of medical supplies based on VOC.

#### 4.1.4. Define the Parameters That Will Be Used to Assess the Cost Level of Products

Determining the parameter that will be used for assessing the cost of medical supplies will be based on item purchasing cost. A simple scale system was used for ranks, such that (5) indicated high cost, (3) medium cost, and (1) low cost.

#### 4.1.5. Assess Performance, Criticality, and Cost by Process Owners or Management

All items at the medical supplies warehouse were assessed according to the “average inventory level” parameter that defines the performance according to VOC. The warehouse keepers (the process owners) classified the items as having high performance, medium performance, and low performance. Also, the keepers estimated the desired targeted performance for each item and measured the gap. High-performance items are items whose average inventory is well matched to the demand. Medium-performance items are items whose average inventory is less matched to demand. While, low-performance items are items whose average inventory is much higher than the quantity needed to cover the demand. A simple scale system was used to rank items in terms of performance level, such that (1) indicated high performance level, (5) medium performance level, and (9) low performance level. Then, depending on the gap between the performance level and desired target level, the level was multiplied by the factor F3, such that the larger the gap, the higher the value of the factor F3. The factor F3 has a value between zero and one.

Based on VOC, two criteria were chosen to be used for assessing the criticality of medical supplies, the criteria are “time needed to get product from the nearest distributor warehouse” and “effect of stock-out incidents or problem caused by stock-out condition.” All items at the medical supplies warehouse were checked for criticality with the process owners (doctors and nurses), then classified into high, medium, or low criticality level, and finally process owners estimated the desired targeted criticality for each item and measured the gap. High-criticality items were those for which a stock-out condition was life-threatening for the patient. Medium-criticality items had less effect on the patient in case of a stock-out but did affect the diagnosis or treatment. Low-criticality items were of almost no effect in case of stock-out except the inconveniences of the patient. A simple scale system was used to rank items in terms of criticality such that (9) indicated high criticality, (5) medium criticality, and (1) low criticality. Then, depending on the gap between the criticality level and desired target level, the level was multiplied by the factor F1, such that the larger the gap, the higher the value of the factor F1. The factor F1 has a value between zero and one.

Cost indicators were based on purchasing cost data that were acquired from the procurement department, and then, process owners estimated the desired targeted cost for each item and measured the gap. A simple scale system was used to rank items in terms of cost such that (9) indicated high cost, (5) medium cost, and (1) low cost. Then, depending on the gap between the cost level and desired target level, the level was multiplied by the factor F2, such that the larger the gap, the higher the value of the factor F2. The factor F2 has a value between zero and one.

The Importance Index was calculated for all items at the medical supplies warehouse depending on the process owners' evaluation of the three levels and the gap from target assessment. As a sample for demonstration in this paper, Table 5 shows the Importance Index calculations for some of the items. Note that the two items with the highest II value among all items were included in the sample in Table 5. These two items are “Intra-aortic balloon” and “Seroquel 300 mg tablet.”

#### 4.1.6. Define Project Goals

The project goal is to reduce the II value for Intra-aortic balloon and Seroquel 300 mg tablet.

### 4.2. Measure Phase

#### 4.2.1. Map Current Process

**Figure 1:**
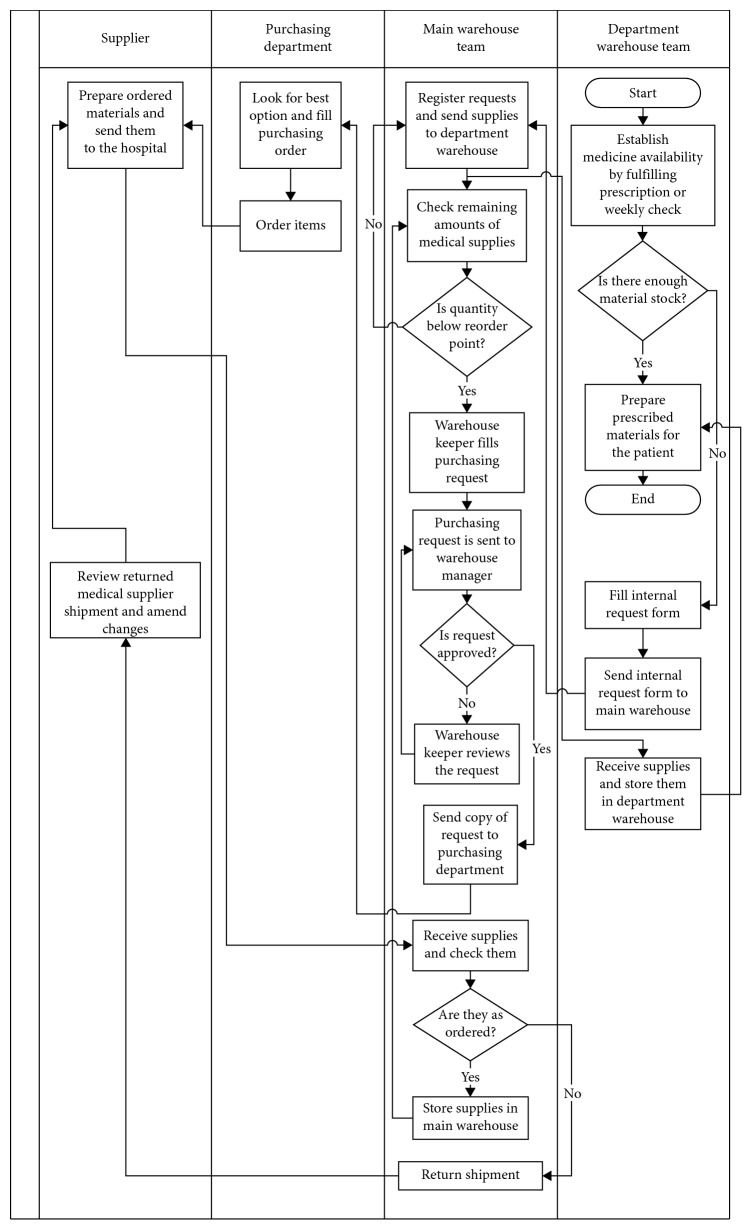
Logistics activities process map at the medical supplies warehouse.

Based on the acquired knowledge in the define phase, the information and material flow chart of the logistics system at the case hospital were developed as shown in Figure 1.

#### 4.2.2. Measure Process Performance Parameters

All items at the medical supplies warehouse were assessed by the Six Sigma project team according to the “average inventory level” parameter that had the highest average CTQ rank as found by VOC and using the same scale system used by the process owners.

#### 4.2.3. Measure Process Criticality Parameters

All items at the medical supplies warehouse were checked for criticality by the Six Sigma project team and then classified into high, medium, or low criticality level using the same scale system used by the process owners.

#### 4.2.4. Measure Process Cost Parameters

The cost of all items at the medical supplies warehouse was assessed by the Six Sigma project team using the same scale system used by the process owners.

#### 4.2.5. Calculate Performance Index

Using the output of the 3 steps above, the levels were set, and factors were then estimated depending on the desired targeted levels provided by process owners in the define phase. The Importance Index was calculated for all items at the medical supplies warehouse by the Six Sigma project team. Again, as a sample for demonstration in this paper, Table 6 shows the Importance Index calculated by the Six Sigma team for the same sample of items in Table 5. As a result of this step, the II values for “Intra-aortic balloon” and “Seroquel 300 mg tablet” are still the highest among all items. Therefore, the project selection done during the define phase is still valid, and the Six Sigma team proceeded the work with the defined project (reduce the II value for Intra-aortic balloon and Seroquel 300 mg tablet).

### 4.3. Analyse Phase

#### 4.3.1. Improve the Process

**Figure 2:**
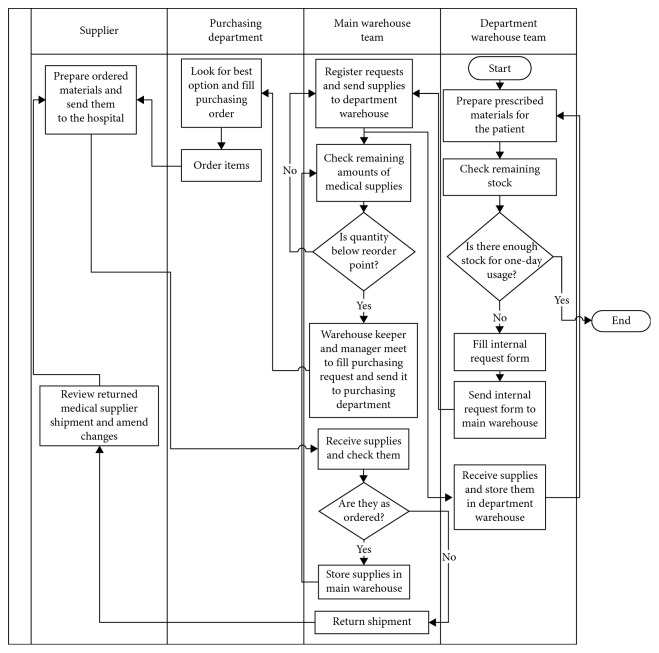
Improved process.

The Six Sigma team carefully observed the as-is process and used value stream analysis to improve it, which included eliminating and minimising nonvalue-added activities, developing and moving inspection points forward, or eliminating them.  Figure 2 shows the improved process. Changes done to reduce the process complexity included:

- The check of the items' quantity on hand is done after giving the medicine to patients rather than a weekly check. This eliminated the possibility of having stock outs as the check is done after each prescription is given.
- Rather than having the main warehouse keeper fill the purchasing request and then send it for the warehouse manager for approval, a meeting is conducted were the warehouse manager approves the requests and then the keeper fills them and sends them to the purchasing department.
- Shipment inspection is done at the supplier side to save time.

#### 4.3.2. Determine Potential Causes for the Problem on Hand

A brainstorming session was held along with the case hospital staff within the related departments (the cardio department staff, warehouse officer, and material management officer) to put down all the possible causes that lead to having such a high II value for the “intra-aortic balloon.” It was agreed that the main cause for such low performance level causing a high II value was the fact that the “intra-aortic balloon” has been replaced by new medications that are easier on the patient and more cost justified. Therefore, the “intra-aortic balloon” became an obsolete item that is to be used only in rare emergency situations when no other medication would save the patient's life.

**Figure 3:**
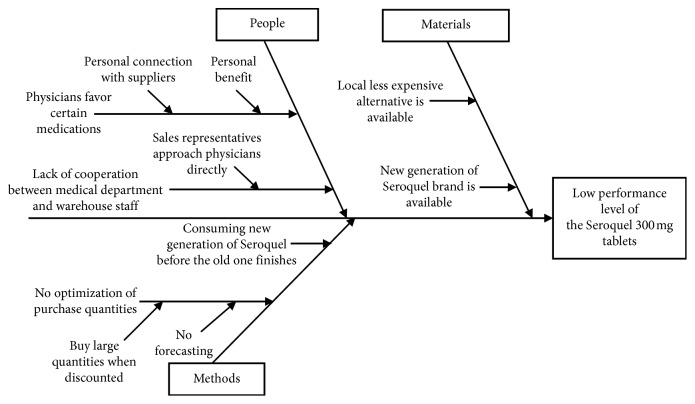
Cause-and-effect diagram showing possible causes of the low performance level for Seroquel 300 mg tablets.

Another brainstorming session was held along with the case hospital staff within the related departments (the neurology department staff, warehouse officer, and material management officer) to put down all the possible reasons that lead to having such a high II value for the “Seroquel 300 mg tablet.” A cause-and-effect diagram was developed to summarise the possible causes of having low performance level that is causing a high II value for the “Seroquel 300 mg tablet” as shown in Figure 3.

The whole picture of this problem developed from further analysis of the causes listed in Figure 3 was summarised as follows:

“Seroquel 300 mg tablet” was the favourite medication for a large number of neurology physicians and the demand for it was high. Since the cost of “Seroquel 300 mg tablet” is rather high, the supplier offered a good bargain based on quantity discount principle. The purchasing officer saw this to be a good chance of saving on the long run and agreed to the supplier's deal. Before the last lot of “Seroquel 300 mg tablet” was consumed, a new generation of the same brand of “Seroquel 300 mg tablet” was available on the market. Because the new generation is an improved one, it became the physicians' new favourite. At almost the same time, a local less-expensive alternative to “Seroquel 300 mg tablet” was available on the market. Since this alternative was less expensive and approved by the Ministry of Health, many physicians switched to prescribing it.

All the above causes combined led “Seroquel 300 mg tablet” to have a low performance level (i.e., its average inventory is much higher than the quantity needed to cover the demand). However, it was agreed that the main cause of this problem is purchasing large quantities of “Seroquel 300 mg tablet” at quantity discount.

#### 4.3.3. Develop Alternative Solutions for the Problem

After studying the cases that led to having a high II value for “Seroquel 300 mg tablet,” the Six Sigma team developed some suggested solutions to reduce the high II value for this item.

One of the suggestions was to set up a “sale representatives' affair office” where his/her main task is to receive offers from sales representatives of medical supplies without representatives approaching physicians directly. This is hoped to guarantee that physicians' choices of medical supplies are purely according to the medical benefits regardless of the supplier-physician relationship.

The rest of improvement options were directly related to the current policies used for material management. One of the suggestions was to set up a policy concerning checking up for new development on current medical supplies or alternatives each time a purchase is done to reflect that on the quantity purchased. Another suggestion was to set up a policy to buy items depending on demand forecasting rather than on offered discounts. The hospital is recommended to conduct demand forecast for all important items stored and order them depending on forecasted demand. The last suggestion was to implement the first-in-first-out (FIFO) method within the warehouse and not to buy new products while still holding considerable stock from it, from its equivalents or from its older version products.

### 4.4. Improve Phase

The new improved process (developed in Section 4.3.1) was implemented at this phase. Also, at this stage and after selecting the alternatives to be implemented for reducing the II index, the Six Sigma team met with the process owners; explained the alternatives, the selection criteria for the best alternative and the selected alternative. The selected alternative was then implemented and the improvement resulted from its implementation was measured. Mainly, the implementation of selected alternative resulted in reducing the gap between the performance level and desired target level which in turn resulted in reducing the II value for both: II for “Intra-aortic balloon” and “Seroquel 300 mg tablet” were reduced by 25% and 33%, respectively. Even though customer satisfaction rates were not compared before and after the project, it is expected to see a marked improvement as well.

### 4.5. Control Phase

Six Sigma control phase ensures that all root causes of the problem seize to exist and that the process will never go back to its old state. For this purpose, a guide with all the suggestions above was created for all warehouses' staff members and for the material management staff and an evaluation system was set to study employees' compliance with the guidelines.

## 5. Conclusions and Future Work

Six Sigma is a pioneer problem-solving technique and a leading process improvement method. We presented how it can be used effectively to deal with healthcare logistics issues, including deciding the major problems to be solved and solving them. We also showed that some tools used for manufacturing applications might not be very useful in service applications such as healthcare logistics without modifications, so we built a framework that could be used to select and implement Six Sigma projects.

This work revealed new contributions on all the published work especially those shown in Table 1 in many ways: First, it reveals a new practical method to select projects based on criticality. Second, it showed how we can include competing performance measures in one calculated measure we called importance index. Third, it revealed a framework that is in line with Six Sigma teachings and with decision maker's priorities.

This article paves the road for research to elaborate on ways to use Six Sigma in the area of improving healthcare logistics, especially that this area of research is almost untouched thus far. This research can be completed and complimented by others who can use this methodology, conduct calculations of the importance factor, check the model validity and implement it in other countries and different types of hospitals, and report case studies in future research.

For hospital management, this work reveals the idea of prioritization of all stocked items based on criticality, cost, and performance. Hospitals should use the II calculations for all items in stock in order to establish current-status review and continuous performance reviews for all items at stocks.

Six Sigma is a management tool. Hospital management may differ from that of a typical company; thus, future research may also reveal obstacles and opportunities when dealing with hospital management while implementing a Six Sigma project in healthcare logistics.

## Figures and Tables

**Table 1:** Overview of important publications.

Publication	Publication approach	Six Sigma implementation focus areas	Six Sigma methodology implemented	Six Sigma tools and techniques used	Six Sigma implementation benefits
Jin et al. [19]	Detailed case study on the application of Six Sigma and Lean in healthcare logistics	Warehouse management	DMADV (define, measure, analyse, design, and verification) methodology and Lean thinking principles	(i) Survey (ii) Critical to quality (CTQ) parameters (iii) Value stream mapping (iv) Fishbone diagram (v) Lean thinking tools (smoothing flow and removing nonvalue-adding activities)	(i) Better storage management (ii) Better use of space (iii) Improved workspace (iv) Organization and cleanliness (v) More timely and efficient delivery of right supplies to the right patients (vi) Tracking and reducing waste (vii) Cost savings

Craven et al. [20]	Brief case studies on the application of Six Sigma and Lean in clinical, operational, and service areas in healthcare	(i) Isolation management (clinical area) (ii) Inventory management (operational area) (iii) Patient room turnaround time (service area)	DMAIC (define, measure, analyse, improve, and control) methodology and Lean thinking principles	(i) Priority ranking for project selection (ii) Voice of customer (VOC) (iii) Survey (iv) Process flow map (v) Lean techniques (sort, straighten, sanitize, standardize, and sustain) (vi) Cause-and-effect analysis	(i) Reduced operating expenses (ii) Reduced patient length of stay (iii) Improved throughput (patient flow) (iv) Compliance and full accreditation (v) Extended expertise (vi) External validation through awards

Lifvergren et al. [21]	Description of lessons learned from the application of Six Sigma projects in different clinical areas in healthcare	Healthcare quality, patient safety, and resource utilization in different clinical areas (one of which is patients' logistics)	DMAIC (define, measure, analyse, improve, and control) methodology		(i) Reducing unwanted variation in care processes (ii) Increased patient safety (iii) Indirect quality improvement (iv) Optimize resource utilization (v) Cost savings

**Table 2:** Proposed framework for applying Six Sigma in healthcare logistics.

Phase	Steps	Description
Define	(1) Fully define the process	This is done by defining the logistics activities performed to obtain the medical product and to ensure its availability, for example, purchasing, transportation, warehousing, and inventory control
	(2) Define the parameters that will be used to assess process performance	Examples of process parameters may include (i) Average inventory level (ii) On-time delivery (iii) Actual time for stock replenishment (iv) Number of stock-out incidents (v) Number of expired holding items (vi) Transporting cost (vii) Actual time to get product from warehouse or store (viii) Number of products damaged in handling or delivery (ix) Volatility and variability of demand (x) Shelf-life (xi) Suppliers reliability (xii) Inventory cost (xiii) Total logistics cost
	(3) Define the parameters that will be used to assess criticality level of the product	Examples of criticality parameters may include (i) Product availability at the nearest distributor/manufacturing warehouse (ii) Time needed to get product from the nearest distributor warehouse (iii) Number of alternative products in the hospital or local market (iv) Effect of stock-out incidents or problems caused by stock-out condition
	(4) Define the parameters that will be used to assess product cost level	Examples of cost parameters may include (i) Purchasing cost (ii) Ordering cost (iii) Holding cost (iv) Transportation cost
	(5) Assess performance, criticality, and cost by process owners or management	(i) Determine current process performance (ii) Determine current process criticality (iii) Determine the cost levels with the help from accounting department (iv) Assess targeted performance, criticality, and cost, and then measure the gap between the current situation and the target (evaluate the F factors) (v) Calculate the Importance Index to select the project with more pain
	(6) Define project goals	(i) Desired improvement to Importance Index value (ii) Any other ancillary goals

Measure	(1) Map current process	This is a team work that may use some of the following tools: (i) Process flow chart (ii) Input/output analysis
	(2) Measure performance parameters	(i) Six Sigma project team studies the as-is process and collects performance parameters data (ii) Plot the collected performance as-is data using simple statistical tools^*∗*^
	(3) Measure criticality parameters	(i) Six Sigma team studies the as-is process and collects criticality parameters data (ii) Plot the collected criticality as-is data using simple statistical tools∗
	(4) Measure cost parameters	(i) Six Sigma team studies the as-is process and collects cost parameters data (ii) Plot the collected cost as-is data using simple statistical tools∗
	(5) Calculate Importance Index	(i) Using targeted performance, criticality, and cost, measure the gap between the current situation and the target (evaluate the *F*s) (ii) Find the II value by the Six Sigma team and verify the project selected was the right one

Analyse	(1) Improve the process	Carefully observe the as-is process and use value stream analysis to improve it, this may include: (i) Eliminate or minimise non-value added activities (ii) Develop and apply standards (iii) Move inspection points forward or eliminate them
	(2) Find root causes affecting criticality, performance and cost	Use tools like Pareto charts or fishbone diagram to determine significant causes responsible for the low performance level, high cost level, and high criticality level
	(3) Develop alternative solutions	Suggest process changes alternatives needed to improve current situation of the criticality, performance, and cost levels

Improve	Implement the new improved process	(a) Study the Importance Index expected enhancement for each alternative (b) Perform risk analysis for each alternative (c) Use prioritization matrix to list features of each alternative. Every feature should have weight related to the item's criticality and cost (d) Implement the best alternative that will have the largest effect on reducing II

Control	Define and implement controls to guarantee the process will not go back to its unhealthy state.	(a) Write quality manuals (b) Set key performance indicators to measure performance and a plan to use them (c) Employees training program to maintain skills and transfer knowledge

^*∗*^Pie chart, histogram, scatter plot, and control chart.

**Table 3:** Suggested criteria and average rank of CTQ questionnaire.

Potential CTQ	Average CTQ rank
Average inventory level	4.6
Inventory holding costs	4.2
Number of expired items	4.2
On-time delivery	4.2
Supplier reliability	4.2
Safety stock level	3.8
Number of stock-out incidents	3.8
Actual time to get product from warehouse	3.8
Effect of redundant purchases	3.0
Variability of demand	2.8
Shelf life	2.6
Transporting costs	1.4

**Table 4:** Findings of VOC used to determine criticality categorization criteria.

Suggested criteria	Average criteria rank
Effect of stock-out incidents or problems caused by stock-out conditions	4.1
Time needed to get the product from the nearest distributer warehouse	4.1
Availability of product in the nearest distributer or manufacturing warehouse	4.0
Ease of acquiring the item during diagnosis or operation from store	3.8
Accessibility of item at store after working hours	3.5
Number of alternative products in the hospital or local market	3.3
Unavailability of item due to maintenance or cleaning	3.0

**Table 5:** Importance Index calculated for sample items.

Item name	F1	Criticality level	F2	Cost level	F3	Performance level	Importance Index (II)
Syringe 5 ml	0.7	5	0	1	0	1	3.5
Crepe bandage 6	0	1	0.6	5	0	1	3
Cannula 20	0.7	5	0	1	0	1	3.5
Umbilical cotton tape	0.7	5	0.6	5	0.5	5	9
Latex gloves	0.7	5	0	1	0.5	5	6
N/S 500 ml IV fluid	1	9	0	1	0.5	5	11.5
Surgical blade 10	0.7	5	0	1	0.5	5	6
Gauze swap 4 × 4	0	1	0	1	1	9	9
*Seroquel 300 mg tablet*	**1**	**9**	**0.6**	**5**	**1**	**9**	**21**
*Intraaortic balloon*	**1**	**9**	**1**	**9**	**1**	**9**	**27**

**Table 6:** Importance Index calculated for sample items.

Item name	F1	Criticality level	F2	Cost level	F3	Performance level	Importance Index (II)
Syringe 5 ml	0.7	5	0	1	0	1	3.5
Crepe bandage 6	0	1	0.6	5	0	1	3
Cannula 20	0.7	9	0	1	0	1	6.3
Umbilical cotton tape	0.7	1	0.6	5	0.5	5	6.2
Latex gloves	0.7	5	0	1	0.5	5	6
N/S 500 ml IV fluid	1	9	0	1	0.5	5	11.5
Surgical blade 10	0.7	5	0	1	0.5	5	6
Gauze swap 4 × 4	0	1	0	1	1	9	9
*Seroquel 300 mg tablet*	**1**	**9**	**0.6**	**5**	**1**	**9**	**21**
*Intraaortic balloon*	**1**	**9**	**1**	**9**	**1**	**5**	**23**

## Data Availability

The data used to support the findings of this study have not been made available because they are confidential to the case study hospital.
